# Genome size estimation of false daisy, cheek weed, pot marigold and marigold

**DOI:** 10.6026/97320630019976

**Published:** 2023-09-30

**Authors:** Akhter Ansari Waquar, Krishna Ram, Ajmal Ali Mohammad, Joongku Lee

**Affiliations:** 1 ICAR-Indian Institute of Vegetable Research, Varanasi-221005, Uttar Pradesh, India; 2 Department of Botany and Microbiology, College of Science, King Saud University, Riyadh 11451, Saudi Arabia; 3 Department of Environment and Forest Resources, Chungnam National University, Daehak-ro, Yuseong-gu, Daejeon, Republic of Korea

**Keywords:** Genome size, false daisy, cheek weed, pot marigold, marigold

## Abstract

We report the genome size estimated using flow cytometry for four closely related species, including false daisy (*Eclipta prostrate*), cheek weed (*Ageratum conyzoides*), pot marigold (*Calendula officinalis*), and marigold (*Tagetes erecta*) belonging to Asteraceae family. The detected genome size for false daisy, cheek weed, pot marigold, and marigold was, 2.435, 3.266, 3.413, and 1.897, Gbp, respectively, while their respective 2C DNA content was 2.5, 3.3, 3.5, and 1.9, pg. The information on genome size presented here will be useful for understanding genomic evolution and will also clear the way for additional genomic research in these species.

## Background:

Important members of Asteraceae family include the false diasy (*Eclipta prostrate*), cheek weed (*Ageratum conyzoides*), pot marigold (*Calendula officinalis*), and marigold (*Tagetes erecta*), the strong, straight and ornamental marigold plant is cultivated as an ordinary nursery plant around the world [1]. Pot marigold, on the other hand, is one of the commonly used medicinal plants in China, India, the United States, and Europe [2]. Another plant under study in this area is cheek weed, an annual herb with straight, branching stems and thin, durable roots; there are numerous well-established restorative uses for cheek weed in numerous nations around the world [3]. Herbal plant species false daisies are an annual plant that is typically found in tropical and subtropical regions of the world and have applications in Ayurveda. In the test, it is sour, warm prickly, and parched. In India, false daisy is commonly referred to as bhangra or bhringaraj. It has been used as a medication to treat fever, male pattern baldness, skin conditions, digestive disorders, and ailments of the respiratory system [4]. Even though some recent molecular investigations have been conducted, relatively little genomic information about these four species is now available. However, fundamental studies like estimating these species' genome sizes will speed up genomics efforts. There has only been one report on flow cytometry-based estimates of the nuclear DNA concentration of *Calendula officinalis* to yet [2]. Estimating the genome size, in term as the "C value," has become a recognised application in a variety of biological experiments. It is essential to comprehending evolution and plant adaptation [5]. Hence, we used flow cytometry to estimate the genome size of four species of the Asteraceae family. Thus, the information on genome size presented here will aid in accelerating genomics work in these species with commercial and medical importance.

## Materials and Methods:

## Plant material:

**Figure 1:**
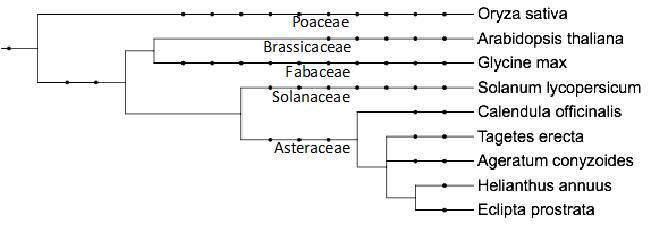
Taxonomical distribution of species including major crops and the four species namely *Eclipta prostrata* (false daisy), *Ageratum conyzoides* (cheek weed), *Calendula officinalis* (pot marigold), and *Tagetes erecta* (Marigold) investigated in the present study to estimate genome size. The estimated genome size data is provided in table 1

The fresh tissues of four species of Asteraceae family, namely, false daisy, cheek weed, pot marigold, and marigold, which is being maintained in Botanical garden of University, were collected and utilized for assessment of genome size using flow cytometric (FCM) technique (Table 1, Figure 1). Fresh young leaves were used since young fast-growing tissues having a high quantity of endopolyploid nuclei, which gives a better outcome. Rice was used as reference control (cv. *Oryza sativa* IR36 with 2C=1.08 pg). Freshly collected leaf tissues were stored in -80°C, and the experiment finished on the same days to decrease the nuclei degradation and other hindrance. Three technical and three biological replicates were taken to reduce the experimental error.

## Flow cytometric analysis:

For the flow cytometric evaluation, LB01 buffer with a final concentration of 15 mM Tris, 2 mM Na2EDTA, 0.50 mM spermine tetrahydrochloride, 80 mM KCl, 20 mM NaCl, and Triton X-1000.10% (vol/vol), was used for nuclei suspension preparation. Fifteen mM β-mercaptoethanol added to the solution before nuclei extraction. Propidium iodide, which intercalates double-stranded DNA was prepared as 100 µg/mL freshly on ice just prior to use (Siga-Aldrich, Germany) [6]. About 100 mg of fresh and young leaf tissues of four Asteraceae species and rice (as a reference) were washed and further processed for sample preparation. Leaf tissues were chopped into small fine pieces in plastic Petri plates with a double-edged sharp razor blade, in 2 mL of ice-cold nuclei isolation buffer. A Corning brand cell strainer (Corning, India) of 40 µm pore size was used to filter the resulting homogenate [7].

## Nuclei staining and flow cytometric data analyses:

Precisely 2 mL of the nuclear suspension of every sample was prepared using 100 mg leaf samples, RNase A (Hi-Media, USA) was added to remove any possible RNA contamination present in the samples; subsequently, 100 µg/mL propidium iodide was added in each sample for staining. To get defined nuclei population's sample were kept in the dark for 1 h [6]. Further analysis was performed in the flow cytometer, Bacton Dickinson FACS LSR-II (BD Biosciences, San Jose, CA, USA). The stained samples were acquired on a flow cytometer using 488 nm Blue and 561 nm Yellow Green lasers to excite propidium iodide and emitted fluorescence signals of propidium iodide was collected on 582/15 bandpass filter. Software BD FACS Diva (version 8.0.1) was used to acquire the samples, and 20000 events were recorded for each sample. Doublet discrimination gate was drawn around the singlets population on PI-W fluorescence (x-axis) vs. PI-A (y-axis) to exclude G0/G1 doublets. To find out the mean fluorescence intensity, G0/G1 peak was selected on the histogram. Based on the huge volume of reports available on FCM related to genome size estimations, the CV limit was set to <5% as measured over 5,000 or 10,000 nuclei content [8]. Further, the standard errors were calculated using the triplicate data [9].

## Results:

## Genome size estimation reference standard:

The selection of appropriate species as a reference standard and its calibration is crucial for accurate genome size estimation with FCM. For any species to serve as an ideal DNA reference standard, its genomic size should be close to that of the target species. Selected rice (*O. sativa*) as a reference in the present study since it is recommended as an ideal standard for genome size estimations. Since rice is a completely sequenced genome with a smaller genome size representing the other end of the spectrum, used it as a reference standard. Present results indicated that rice was found suitable reference standard due to its non-linearity and overlapping spectral issue.

## Estimation and comparison of nuclei content:

**Figure 2:**
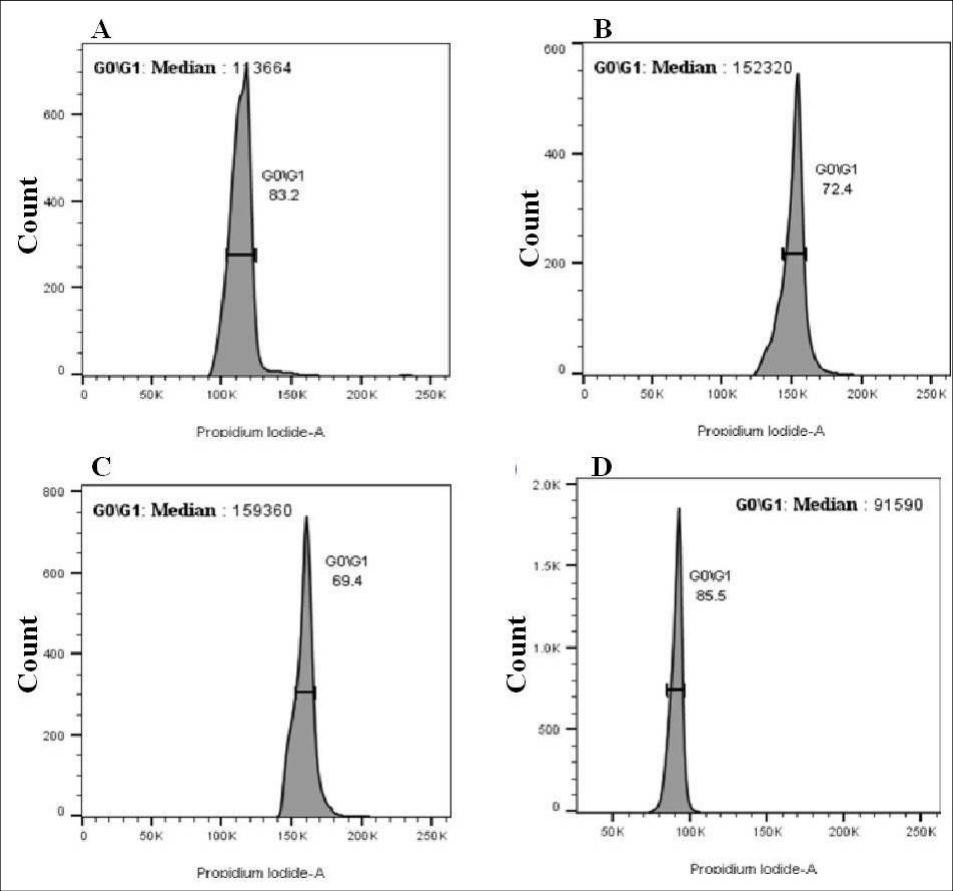
Histograms of PI (Propidium iodide) fluorescence intensity in four species of Asteraceae, namely, (A) *Eclipta prostrata* (false daisy), (B) *Ageratum conyzoides* (cheek weed), (C) *Calendula officinalis* (pot marigold), and (D) *Tagetes erecta* (Marigold). Rice was used as an external standard.

The number of stained nuclei in the test sample was estimated based on the external control and propidium iodide (PI) as the fluorochrome. Clearly defined histograms were obtained for flow cytometric analysis of nuclear DNA content of species, namely marigold, pot marigold, cheek weed, and false diasy. MFI value of nuclei of three replicates (leaf tissue) using rice as an external control was found to be 91590, 159360, 152320, and 113664 (Figure 2A-D), respectively, for marigold, pot marigold, cheek weed, and false diasy.

## Evaluation of genome size of four species of Asteraceae family:

Leaf tissues DNA content variation recorded in marigold, pot marigold, cheek weed, and false diasy. The estimated DNA content (2C) was 1.90 pg, 3.50 pg, 3.30 pg, and 2.50 pg respectively for marigold, pot marigold, cheek weed, and false diasy. It was observed that two species viz: pot marigold and cheek weed showed much close genome content. Among the four species, pot marigold was with maximum genome content, while marigold, with minimum genome content. Thus, from the 2C measurements obtained for the marigold, pot marigold, cheek weed, and false diasy from leaf tissues, their genome size was recorded as 1.897 Gbp, 3.413 Gbp, 3.266 Gbp, and 2.435 Gbp respectively (Table 1).

## Discussion:

Flow cytometry has been considered as one of the standard procedures for estimation of genome size in large number of plant species. This methodology has been used to estimate genome size of various plant species, for instance, *Dipsacoideae* [10]; *Avena* [11]; three genus of Zingiberoideae i.e., *Curcuma*, *Hedychium* and *Kaempferia* [12]; sweet wormwood (*Artemisia annua*) [13]; and Chinese date (*Ziziphus jujuba*) [14]. In spite of the fact that these species have restorative, and numerous other economic significance still the genome size and other genomic data's are as yet constrained for many. Considering the enormous application and reliable outcomes, the flow cytometer based genome size estimation method was used in the present investigation.

Flow cytometry estimation is performed to quantify the DNA content (G0/G1 cell cycle stage) in leaf tissues of the four Asteraceae family plant species, as previously performed in several different plant species [15]. In the common protocol of FCM, plant tissues used instantly after harvesting as freezing of these tissue decreases the FCM histograms separation. Previous information proposes wide variation among flowering plants genome size (> 2500-fold) ranging from 0.06 pg in corkscrew plants (*Genlisea margaretae*) to 152.23 pg in Kinugasaso (*Paris japonica*) [16]. Even within the same species tissues about two-fold variations have been recorded. Within a genus 3-fold variation was reported, with the highest differences of up to 63-fold, this is largely due to the dissimilarity in ploidy levels among the species [17]. In the present study, among four species maximum 1.80-fold variation between two species genome size (2C DNA content) (i.e. marigold with genome size 1.94 pg and pot marigold with the genome size 3.49 pg; Table 1) was recorded. Genome size (Mbp) of pot marigold and cheek weed were found different. Findings indicated that four species studied have genome level variation, though, the genome size difference among the species *Calendula officinalis* and *Ageratum conyzoides* were less significant, which point out their closed diversification to each other. Many regions have been designated by different researchers for genome size inconsistency, like, due to difference in heterochromatin regions and variation in intron size [18], and various other features, which include the copy number of transposable elements (TEs), the pseudogenes number and the amount or size of microsatellite regions, [19]. In addition, differences in TE structures, particularly, that of long terminal repeats may be a factor of genome size variations which have an extensive influence on plant evolution [20]. The genome size data produced in this investigation will be useful in deciding strategy for whole-genome sequencing of these species.

## Conclusion:

Presented result proposes considerable inter-specific difference exists amongst the species studied. Further, it indicates that the species distributed in a diverse climatic situation and acquiring big distribution areas usually have higher variation in their genome sizes and in various traits of genetic importance. The genome size information will be helpful to understand genome plasticity and evolution in the marigold clad and to speed-up the genomics efforts.

## Funding:

Authors extend their appreciation to the Researchers Supporting Project Number (RSP2023R306), King Saud University, Riyadh, Saudi Arabia.

## Figures and Tables

**Table 1:** Genomic DNA content and genome size of four species of Asteraceae family

**Species name**	**Genome size (2C) in pico gram (pg)**	**Genome size in Mbp**
*Ecliptaprostrata* (false daisy)	2.49 ± 0.07^b^	2435.22 ± 41.13^b^
*Ageratum conyzoides*(cheek weed)	3.34 ± 0.09^a^	3266.52 ± 53.22^a^
*Calendula officinalis*(pot marigold)	3.49 ± 0.10^a^	3413.22 ± 65.22^a^
*Tageteserecta*(Marigold)	1.94 ± 0.03^c^	1897.32 ± 29.13^c^

## References

[1] Nguyen DTC (2021). Sci Tot Environ..

[2] Byrne D (2022). Ecol Evol..

[3] Shepherd A (2022). Phytomedicine.

[4] Loeuille B (2021). Peer J..

[5] de Boer HJ (2016). New Phtol..

[6] Dolezcaronel J (2007). Nat. Protoc..

[7] Galbraith DW (1983). Science.

[8] Bainard JD, Newmaster SG. (2010). J. Bot..

[9] Misra R (2000). Y.R. J. Essent. Oil. Res..

[10] Frajman B (2015). BMC Evol. Biol..

[11] Yan H (2016). Genome.

[12] Basak S (2017). Acta Physiol. Plant..

[13] Liu (2018). Plant Biol. Crop. Res..

[14] Wang L (2019). Forests.

[15] Rewers M, Sliwinska E. (2012). Cytometry Part A.

[16] Bennett MD, Leitch IJ. (2011). Ann. Bot.

[17] Grover CE (2008). Mol. Biol. Evol..

[18] Silva JC (2016). PLoS One.

[19] Petrov DA (2001). Trends Genet..

[20] Rabinowicz PD, Bennetzen JL. (2006). Curr. Opin. Plant Biol..

